# Genetically dissecting *P2rx7* expression within the central nervous system using conditional humanized mice

**DOI:** 10.1007/s11302-016-9546-z

**Published:** 2016-11-17

**Authors:** Michael W. Metzger, Sandra M. Walser, Fernando Aprile-Garcia, Nina Dedic, Alon Chen, Florian Holsboer, Eduardo Arzt, Wolfgang Wurst, Jan M. Deussing

**Affiliations:** 1 Max Planck Institute of Psychiatry, 80804 Munich, Germany; 2 Instituto de Investigación en Biomedicina de Buenos Aires (IBioBA)-CONICET- Partner Institute of the Max Planck Society, Buenos Aires, Argentina; 3 Max Planck Institute of Immunbiology and Epigenetics, 79108 Freiburg, Germany; 4 Helmholtz Zentrum München, German Research Center for Environmental Health, Institute of Developmental Genetics, 85764 Neuherberg, Germany; 5 HMNC Brain Health, 80539 Munich, Germany; 6 German Center for Neurodegenerative Diseases (DZNE), Site Munich, 81377 Munich, Germany; 7 Munich Cluster for Systems Neurology (SyNergy), Adolf-Butenandt-Institut, Ludwig-Maximilians-Universität München, 80336 Munich, Germany; 8 Department of Neurobiology, Weizmann Institute of Science, 7610001 Rehovot, Israel; 9 Chair of Developmental Genetics c/o Helmholtz Zentrum München, Technische Universität München-Weihenstephan, 85764 Neuherberg, Germany

**Keywords:** P2X7 receptor, P2rx7 gene, Pore formation, Mouse model, Knockout, Gene expression

## Abstract

The purinergic P2X7 receptor (P2X7R) has attracted considerable interest as a potential target for various central nervous system (CNS) pathologies including affective and neurodegenerative disorders. To date, the distribution and cellular localization of the P2X7R in the brain are not fully resolved and a matter of debate mainly due to the limitations of existing tools. However, this knowledge should be a prerequisite for understanding the contribution of the P2X7R to brain disease. Here, we generated a genetic mouse model by humanizing the P2X7R in the mouse as mammalian model organism. We demonstrated its functionality and revealed species-specific characteristics of the humanized receptor, compared to the murine ortholog, regarding its receptivity to activation and modulation by 2′,3′-O-(benzoyl-4-benzoyl)-adenosine 5′-triphosphate (BzATP) and trifluoperazine (TFP). This humanized *P2rx7* allele is accessible to spatially and temporally controlled Cre recombinase-mediated inactivation. In contrast to previously generated knockout (KO) mice, none of the described *P2rx7* splice variants evade this null allele. By selective disruption and assessment of human *P2RX7* expression in different brain regions and cell types, we were able to demonstrate that the P2X7R is specifically expressed in glutamatergic pyramidal neurons of the hippocampus. Also, P2X7R is expressed in major non-neuronal lineages throughout the brain, i.e., astrocytes, oligodendrocytes, and microglia. In conclusion, this humanized mouse model provides the means for detailed assessment of human P2X7R function in vivo including evaluation of agonists or antagonists. In addition, this conditional allele will enable future loss-of-function studies in conjunction with mouse models for CNS disorders.

## Introduction

P2X receptors are trimeric receptors composed of 3 subunits that can form homo- or heteromers. Each subunit consists of an intracellular C- and N-terminal domain as well as 2 transmembrane domains, joined by a cysteine-rich ectodomain that binds adenosine triphosphate (ATP) [1, 2]. Within the P2X family, P2X7 is the largest subunit consisting of 595 amino acids (aa) with a unique intracellular C-terminal domain of 239 aa, which is significantly longer than in the rest of the family [3–5]. This long C-terminal domain comprises several protein and lipid binding motifs as well as a cysteine-rich domain, including a binding domain for lipopolysaccharides (LPS) [6]. In contrast to other family members, P2X7 receptors are predominantly homomers [7]. Despite the fact that human and murine P2X7 share about 80 % sequence homology, differences between the species have been described with regards to receptor sensitivity toward various ligands. The human P2X7R has been shown to be 10–100 times more sensitive to stimulation by the agonist 2′,3′-O-(benzoyl-4-benzoyl)-adenosine 5′-triphosphate (BzATP) compared to the murine ortholog [4, 8, 9]. Moreover, it has been demonstrated that human and murine receptors show different susceptibility regarding modulation of their activity by various compounds [9–11].

The human *P2RX7* gene is located on chromosome 12 and mouse *P2rx7* on chromosome 5 in a region of conserved synteny. Both genes comprise 13 exons, which give rise to multiple splice variants. While 13 transcripts have been described for human *P2RX7*, only 5 alternative transcripts have been described for the mouse so far: *P2rx7*-*a*, *P2rx7*-*b*, *P2rx7*-*c*, *P2rx7*-*d*, and *P2rx7*-*k* [7, 12, 13] (compare Fig. 4a). *P2rx7*-*b*, *P2rx7*-*c*, and *P2rx7-d* are characterized by a truncated C-terminus. In addition, the first exon in *P2rx7*-*c* and *P2rx7*-*d* is affected by alternative splicing [14]. It has repeatedly been shown that in particular, the C-terminally truncated isoforms have a negative regulatory effect on receptor function when co-expressed with full-length P2X7 [3, 12, 15, 16]. *P2rx7*-*k* is characterized by an alternative exon 1, whereas exons 2–13 are identical to *P2rx7*-*a*. This isoform seemingly does not disturb receptor function; on the contrary, it shows higher sensitivity toward the endogenous agonist ATP. Further, it has been shown that different to the most abundant isoform *P2rx7*-*a*, *P2rx7*-*k* is highly sensitive to activation by extracellular nicotinamide adenine dinucleotide (NAD+) via ADP-ribosylation [17]. Moreover, these 2 isoforms are differentially expressed; *P2rx7*-*k* predominantly occurs in regulatory T cells whereas *P2rx7*-*a* is the dominant variant in peritoneal macrophages and skeletal muscle [13].

The P2X7R is broadly expressed in immune cells of the hematopoietic lineage including monocytes, lymphocytes, macrophages, and dendritic cells [18, 19]. Surprisingly, the precise expression of the P2X7R in the brain is still a matter of debate in the field [20]. In particular, neuronal expression of the P2X7 receptor has been controversially discussed and contested [21, 22]. The non-selectivity of available P2X7R antibodies in the brain has been demonstrated using P2X7R KO mouse lines generated by GlaxoSmithKline and Pfizer, respectively [23, 24]. While the loss of P2X7R protein was readily detected in peripheral tissues, the detection of P2X7R in the brain in both KO lines was masked by an unknown protein of similar size [25, 26]. This finding prevented the reliable detection of P2X7R in the brain including more detailed analyses of spatial receptor distribution in particular brain regions, cell types, or subcellular structures. In addition, it became clear that the KO allele of the GlaxoSmithKline mice is not a complete null allele because splice variant *P2rx7-k* evades inactivation [7, 27]. Similarly, there is evidence that the Pfizer mice are still able to express a C-terminally truncated and at least partially functional P2X7R due to the presence of splice variants *P2rx7-b* and *P2rx7-c* [12, 26, 28]. Thus, the protein detected in the brain of P2X7R KO mice might represent a *P2rx7* splice variant that evades inactivation. For the most recent P2X7R KO mice, generated by Lexicon Pharmaceuticals and the European Conditional Mouse Mutagenesis (EUCOMM) program, respectively, no information with respect to splice variants has been provided so far [29]. Similarly, the presence of *P2rx7* splice variants has not been evaluated in P2X7R knockdown mice generated by transgenic siRNA technology [30].

The shortcomings and uncertainties in the P2X7R field specified above are in sharp contrast to the increasing attention the P2X7R has gained in recent years as an emerging target in particular for CNS diseases [31, 32]. Therefore, the aim of this work was to overcome major obstacles in P2X7R research by providing the following: (i) an in vivo system to test the properties of human P2X7R, (ii) a mouse line that possesses a complete null allele lacking all currently known splice variants, and (iii) a genetic tool to assist the localization of the P2X7R in the CNS.

## Materials and methods

### Generation of humanized P2X7R mice

Humanized P2X7R (*hP2RX7*) mice were generated by knock-in of human *P2RX7* cDNA to the murine *P2rx7* locus. The homology arms of the targeting vector (amplified by PCR from genomic DNA of TBV2 (129S2/Sv) embryonic stem (ES) cells) enframe from 5′ to 3′: a *loxP* site followed by the 3′-end (1.4-kb) of mouse intron 1; the murine exon 2 is replaced by the human *P2RX7* cDNA comprising exons 2–13; a reverse oriented selection marker flanked by *frt* sites which consists of a phosphoglycerate kinase (PGK) promoter driven neomycin resistance gene equipped with a bovine growth hormone (bGH) poly A signal (pA), a second *loxP* site and a quadruple poly A signal consisting of a bGHpA, a PGK pA, and 2 SV40 pAs. The full-length human P2X7R cDNA was amplified by PCR from human hippocampus cDNA using primers: forward: 5′-CAC-CAT-GCC-GGC-CTG-CTG-CAG-CTG-CAG-TGA-TGT-TTT-3′ and reverse: 5′-GTA-AGG-ACT-CTT-GAA-GCC-ACT-GTA-CTG-CCC-TTC-ACT-3′ [33]. This cDNA appeared with the following amino acid sequence at the 11 positions of previously described haplotypes P2X7–1, P2X7–2, and P2X7–4: Val-76, Gly-150, His-155, Arg-270, Arg-276, Arg-307, Ala-348, Thr-357, Gln-460, Glu-496, Ile-568 [34].

Mutant ES cells were used to generate chimeric mice by blastocyst injection. Germ-line transmission of the modified *P2rx7* allele (*P2rx7* ^*hP2RX7-neo*^) was confirmed in offspring from male chimeras bred to wild-type C57BL/6N mice. Finally, the *frt* flanked selection cassette was removed by breeding to *Deleter-Flp* mice [35]. Mice with a humanized *P2rx7* allele (*P2rx7* ^*hP2RX7*^) with conditional potential were kept on a mixed 129S2/Sv × C57BL/6N background.

### Generation of conditional P2X7R knockout mice

Conditional P2X7R KO mice were generated by breeding *hP2RX7* mice to specific Cre drivers. Heterozygous *P2rx7* ^*+/hP2RX7*^ Cre positive mice were either directly used for analysis by RT-qPCR or further bred to generate homozyogous *P2rx7* ^*hP2RX7/hP2RX7*^ Cre positive mice, which were used for preparation of primary cultures or in situ hybridization.

The following Cre drivers were used: *Deleter-Cre*, Cre expression driven by the ubiquitous *Rosa26* promoter (purchased from TaconicArtemis, Cologne, Germany); *Nes-Cre*, Cre expression driven by nestin promoter, which covers neurons and macroglia of the CNS [36]; *Nex-Cre*, Cre expression in forebrain glutamatergic neurons [37]; *Dlx5/6-Cre*, Cre-mediated recombination in forebrain GABAergic neurons [38]; *Glast-CreERT2*, expression of tamoxifen-inducible Cre in astrocytes [39]; *Cnp-Cre*, Cre expression in oligodendrocytes [40]; *Cx3cr1-CreERT2*, expression of tamoxifen-inducible Cre in microglia [41]; *En1-Cre*, expression of Cre recombinase in neurons and macroglia of the mid/hindbrain boundary [42]; *Alpha6-Cre*, expression of Cre recombinase under the control of the promoter of the GABA A receptor, subunit alpha 6 in granule cells of the cerebellum [43]. For all experiments involving inducible Cre recombinase lines, tamoxifen was administered via food pellets (LAS CRdiet CreActive TAM400, LASvendi) for 2 weeks.

### Genotyping

Genotyping was performed by PCR using primers: hP2RX7-mIntron1-for 5′-AGA-CTC-TCA-CCA-GCA-GCA-GCT-C-3′, hP2RX7-hExon6–7-rev 5′-CAG-GAT-GTT-TCT-CGT-GGT-GTA-G-3′, hP2RX7-mIntron2-rev 5′-GCC-AAG-CAT-TCT-ACC-AGT-TGA-GC-3′, hP2RX7-KO-for 5′-GCA-GTC-TCT-CTT-TGC-CTC-GT-3′, hP2RX7-KO-rev 5′-CGT-CGA-CTG-TCT-TCT-GGT-CA-3′ resulting in a wild-type PCR product of 417 bp, a 613 bp product for the floxed humanized allele and a 222 bp product for the KO allele.

### Animals and animal housing

All mice were housed under standard laboratory conditions and were maintained on a 12-h light-dark cycle (lights on from 7:00 am to 7:00 pm), with food and water provided ad libitum. All animal experiments were conducted in accordance with the Guide for the Care and Use of Laboratory Animals of the Government of Upper Bavaria, Germany as well as with the Animal Care and Use Committee of the Max Planck Institute of Psychiatry (Munich, Germany).

### Primary cell culture

Primary neuronal cultures were prepared using mouse embryos 18 days post-coitum as previously described [44]. Astrocytic, microglial, and mixed cultures were prepared from mice at postnatal day 2. For neuronal cultures, pregnant mothers were sacrificed by an overdose of isoflurane and embryos by decapitation; for other cultures, pups were sacrificed by decapitation. For all cultures, the brains were dissected free of meninges. Cortices and hippocampi were isolated together, subsequently dissociated, and suspended in respective growth media. Neurons were grown in neurobasal-A medium supplemented with B27 Supplement (Invitrogen) and GlutaMAXI (Invitrogen). Astrocytes were grown in DMEM (Invitrogen) supplemented with 10 % FCS and 1 % penicillin/streptomycin, microglia, and mixed cultures in DMEM/F12 (Invitrogen) also supplemented with 10 % FCS and 1 % penicillin/streptomycin. To isolate astrocytes and microglia from mixed cultures, cells were trypsinized when confluency was reached until the astrocyte containing cell layer detached. To enrich astrocytes, the floating cell layer was dissociated and transferred to a new plate [45]. For microglia-enriched cultures, the floating cell layer was aspirated and replaced by fresh media as previously described [46].

### Reverse transcriptase quantitative real-time PCR (RT-qPCR)

For quantification of mRNA expression levels, RNA was isolated using TRIzol (Invitrogen) and transcribed to cDNA using the *SuperScript II Reverse Transcriptase Kit* (Invitrogen) following the manufacturer protocols. Then, qPCR was carried out in a LightCycler96 (Roche) using the *SYBR Green I Master*–kit (Roche). The following primers for P2X family members and markers for specific cell types were used: P2rx1-for 5′-TAT-CCT-TGT-GGA-TGG-CAA-GG-3′, P2rx1-rev 5′-TCT-TAG-GCA-GGA-TGT-GGA-GC-3′, P2rx2-for 5′-CGT-GTG-GTA-CGT-CTT-CAT-CG-3′, P2rx2-rev 5′-TGG-CAG-GTA-GAG-CTG-TGA-AC-3′, P2rx3-for 5′-ACA-AGA-TGG-AGA-ATG-GCA-GC-3′, P2rx3-rev 5′-GCA-GGA-TGA-TGT-CAC-AGA-GAA-C-3′, P2rx4-for 5′-GAC-CAA-CAC-TTC-TCA-GCT-TGG-3′, P2rx4-rev 5′-GTG-ACG-ATC-ATG-TTG-GTC-ATG-3′, P2rx5-for 5′-GCC-TAT-ACC-AAC-ACC-ACG-ATG-3′, P2rx5-rev 5′-CTT-CAC-GCT-CAG-CAC-AGA-TG-3′, P2rx6-for 5′-GTT-AAG-GAG-CTG-GAG-AAC-CG-3′, P2rx6-rev 5′-AGG-ATG-CTC-TGG-ACA-TCT-GC-3′, P2rx7-for3 5′-CTG-GTT-TTC-GGC-ACT-GGA-3′, P2rx7-rev3 5′-CCA-AAG-TAG-GAC-AGG-GTG-GA-3′, hP2RX7-for3 5′-ATG-TCA-AGG-GCC-AAG-AAG-TC-3′, hP2RX7-rev3 5′-AGG-AAT-CGG-GGG-TGT-GTC-3′, LC_mouse Exon1-for 5′-CAC-ATG-ATC-GTC-TTT-TCC-TAC-3′, LC_mouse Ex2-rev2 5′-CCC-TCT-GTG-ACA-TTC-TCC-G-3′, LC_human Ex2-rev2 5′-TTC-TCC-ACG-ATC-TCC-TCT-T-3′, mRPL19-for 5′-GCA-TCC-TCA-TGG-AGC-ACA-T-3′, mRPL19-rev 5′-CTG-GTC-AGC-CAG-GAG-CTT-3′, GFAP-for 5′-ACC-AGC-TTA-CGG-CCA-ACA-G-3, GFAP-rev 5′-CCA-GCG-ATT-CAA-CCT-TTC-TCT-3, CathepsinS-for 5′-CCA-TTG-GGA-TCT-CTG-GAA-GAA-AA-3′, CathepsinS-rev 5′-TCA-TGC-CCA-CTT-GGT-AGG-TAT-3, Synaptophysin-for 5′-AGT-GCC-CTC-AAC-ATC-GAA-GTC-3, Synaptophysin-rev 5′-CGA-GGA-GGA-GTA-GTC-ACC-AAC-3.

### Analysis of splice variants

Splice variants were detected by nested RT-PCR with specific primer combinations for the respective splice variants (Fig. 4a, Table 1).

**Table 1:** RT-PCR primers used to specifically detect different *P2rx7* splice variants

Primers for first PCR	Exon	Code	Sequence 5′ → 3′
Exon1-for	1	A	CGCCCCCGCTGCAGTCACTG
Exon13-rev	13	B	CGTGGAGAGATAGGGACAGCC
Exon12-for	12	E	CGTTGAAGTATGTGTCCTTTGTCG
Exon13b-rev	13b	F	TTCTTAAATAAATGAATTGAAATCAAG
Exon4-for	4	I	TGCTCTTCTGACCGGCGTTG
Exon13c-rev	13c	J	TCAGGTGCGCATACATACATG
Exon1c-for	1c	M	CACATGATCGTCTTTTCCTAC
Exon4b-rev	4b	N	TGACCATTCTCCTGGCTGAC
Exon1d-for	1d	Q	GCCCGTGAGCCACTTATGC
Exon5-rev	5	R	CCTTGTCTTGTCATATGGAAC
Primers for nested PCR			
Exon1-for2	1	C	CCGCTGCAGTCACTGGAG
Exon13-rev2	13	D	GTCGGAGAAGTCCATCTGGGGTC
Exon12-for2	12	G	GCATGGTGGACCAGCAGCTGC
Exon13b-rev2	13/13b	H	CAGTCGTCCAGGAAGTCAGCCG
Exon9-for	9	K	GAGAACAATGTGGAAAAGCGG
Exon13c-rev2	13c	L	CATGCAGGCAAAGCACCCGTAC
Exon2-for	2	O	TGGTGAGCGATAAGCTGTAC
Exon4b-rev2	4b	P	CAAGTATCTGCCTCCCTTTTGAGC
Exon1d-for2	1d	S	GCATATGGATCGGGACGCTGAAG
Exon4-rev	4	T	GGTCAGAAGAGCACTGTGC

For localization of primers, see Fig. 4a

### Calcium imaging

Cells were loaded for 45 min in darkness with Fluo-4 AM 6 μM (Molecular Probes) and Pluronic F-127 0.14 % (Molecular Probes) in a Ca^2+^-buffer (125 mM NaCl, 5 mM KCl, 0.4 mM CaCl_2_, 1 mM MgSO_4_, 5 mM NaHCO_3_, 1 mM Na_2_HPO_4_, 10 mM glucose, 20 mM Hepes, pH 7.4), and then placed on the stage of a fluorescence Olympus IX81 inverted confocal microscope or a Tecan Genios Pro (Tecan) plate reader. Microscope pictures were captured with the 10× UPlanSApo (0.4 numerical aperture) objective, and cells were plated on 8-well culture slides (Nunc Lab-Tek II Chamber Slide/Thermo Scientific). Plate reader experiments were conducted in black 96 well plates (Nunc/Thermo Scientific). Calcium imaging data are presented as ∆F/F_o_, where F_o_ is the resting fluorescence (before stimulation) and ∆F is the peak change in fluorescence from resting levels.

### Yo-Pro-1-upake assay

The Yo-Pro-1-uptake assay was conducted on the plate reader Tecan Genios Pro (Tecan). Cells were plated in black 96-well plates (Nunc/Thermo Scientific). Prior to the experiment, culture medium was carefully aspirated, and Yo-Pro-1-assay buffer (5 mM KCl, 0.5 mM CaCl_2_, 280 mM sucrose, 10 mM glucose, 10 mM hepes, pH to 7.4) with 1 μM Yo-Pro-1 (and 3 μM TFP) was applied. Measurement was immediately started, and after acquisition of a basal value, BzATP in the respective concentration was applied.

### Western blotting

For detection of P2X7 via Western blot, fresh tissue was homogenized, lysed, and subsequently analyzed by SDS-PAGE followed by immunoblotting using antibodies against the C-terminal domain of P2X7R (Alomone Labs, Cat no APR-004; 1:1000) and β-actin (Cell Signaling, Cat no 4967; 1:2000).

### In situ hybridization

For in situ hybridization, ^35^S–UTP-labeled riboprobes were hybridized on 25-μm-thick brain cryosections. The mouse-specific *P2rx7* probe comprises nucleotides 1215–1636 of GenBank accession no. NM_011027. The human-specific *P2RX7* probe comprises nucleotides 1195–1616 of Genbank accession no. NM_002562.

### Interleukin 1β assay

Peritoneal macrophages were isolated as previously described (Basso et al. 2009). A total of 3 μg/ml of LPS was added, and the cells were allowed to prime for 2 h. Subsequently, the cells were challenged with 1 mM of the P2X7R agonist BzATP for 30 min. Supernatants were collected and analyzed for interleukin 1 beta (IL-1β) using an enzyme-linked immunosorbent assay (ELISA) kit following the manufacturer’s instructions (Endogen, Pierce Technology, Rockford, IL, USA).

### Statistical analysis

Data and statistical analyses were performed with the computer programs GraphPad Prism 5.0 (GraphPad software Inc., La Jolla, CA) and SPSS 16.00 (SPSS Inc., Chicago, IL). All results are shown as means ± standard error of the mean (SEM). Simple comparisons were evaluated with Student’s *t* test (two-tailed). Time-dependent measures were assessed with multi-factorial analysis of variance (ANOVA) with repeated measures (RM-ANOVA). The effects of genotype and/or treatment on IL-1β release and calcium uptake were assessed by two factorial analysis of variance (two-way ANOVA). Whenever significant main or interaction effects were found by the ANOVAs, Bonferroni post hoc tests were carried out to locate simple effects. Statistical significance was defined as *p* < 0.05. *p* values between 0.05 and 0.1 were reported as trends.

## Results

### Establishment of humanized P2X7R mice

**Fig. 1:**
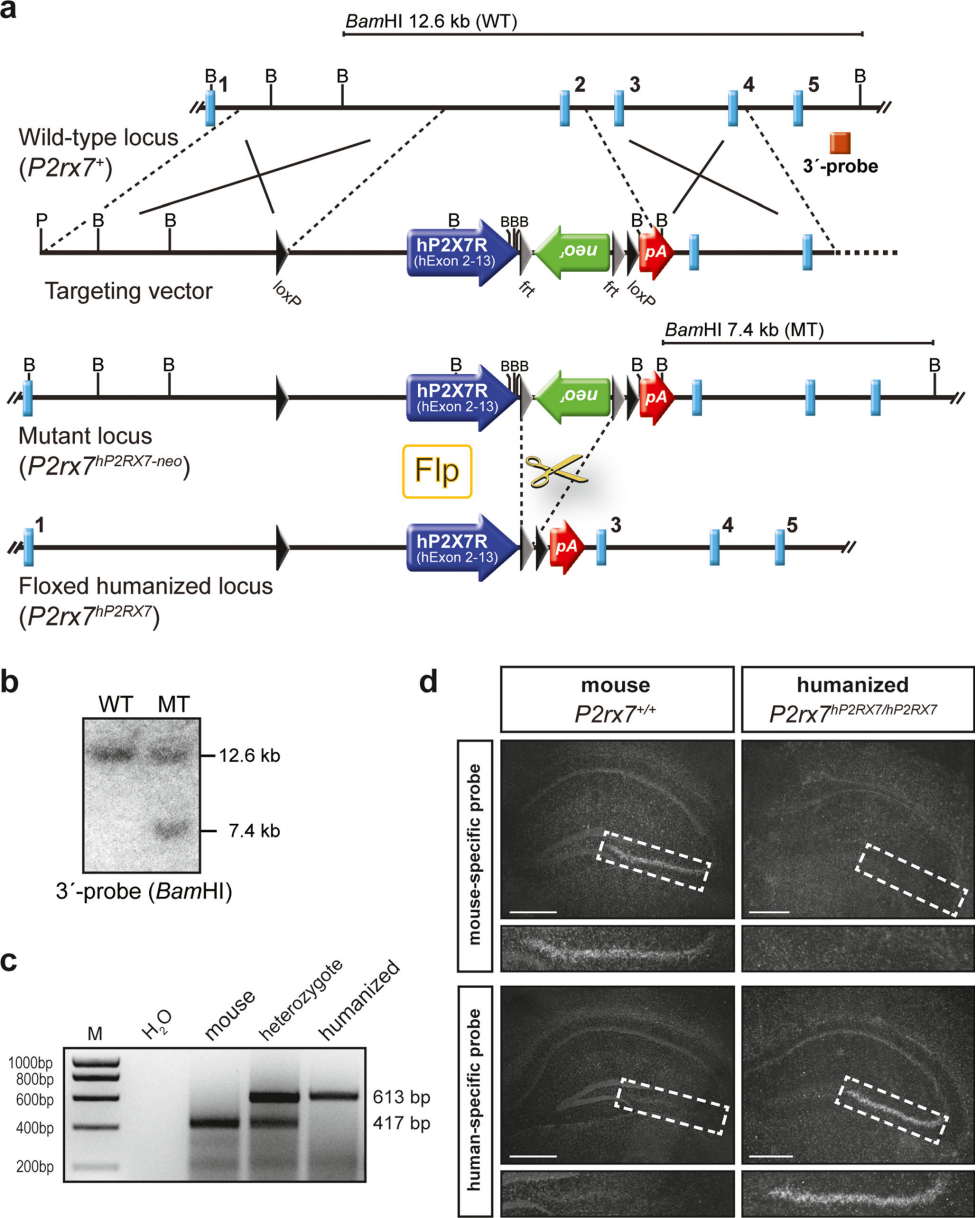
Generation of humanized *P2RX7* (*hP2RX7*) mice. **a** Strategy for knock-in of the human *P2RX7* cDNA. Partial restriction maps of the wild-type *P2rx7* locus, targeting vector, mutant locus following homologous recombination and *loxP* flanked (floxed) humanized locus after Flp recombinase-mediated deletion of the selection marker (B, *Bam*HI; pA, polyadenylation signal). **b** Southern blot analysis of wild-type and targeted ES cell clones. The 3′-probe was hybridized to *Bam*HI-digested genomic ES cell DNA carrying the construct for humanizaton. The targeted allele is indicated by the presence of an additional 7.4-kb fragment. **c** Genotyping was performed by PCR resulting in a 417 bp product for the murine wild-type allele and a 613 bp product for the humanized allele. **d** The expression of human *P2RX7* in knock-in mice recapitulates endogenous expression of murine *P2rx7* as demonstrated by in situ hybridization with riboprobes specific for mouse and human transcripts, respectively. A magnification of the hippocampal CA3 subfield is shown below each overview of the hippocampus. *Scale bars* indicate 500 μm

To study the human P2X7 receptor (hP2X7R) in an in vivo context, we generated a mouse line that expresses the hP2X7R under the regulatory control of the endogenous murine *P2rx7* locus (Fig. 1a–c). To ensure that the temporal and spatial expression of the hP2X7R variant is indistinguishable from the endogenous mouse P2X7R expression, we applied a knock-in strategy substituting mouse exon 2 by the human cDNA covering exons 2–13. The correct expression of the humanized P2X7R was confirmed by in situ hybridization in the hippocampus using species-specific riboprobes. In particular, the prominent expression in the cornu ammonis subfield 3 (CA3) was readily detectable (Fig. 1d). Moreover, this approach guaranteed that the expression level of the humanized P2X7R was identical to the endogenous murine P2X7R, enabling a meaningful direct comparison of murine and humanized receptors when comparing wild-type and humanized mice.

### Comparison of activities of humanized and mouse P2X7R

**Fig. 2:**
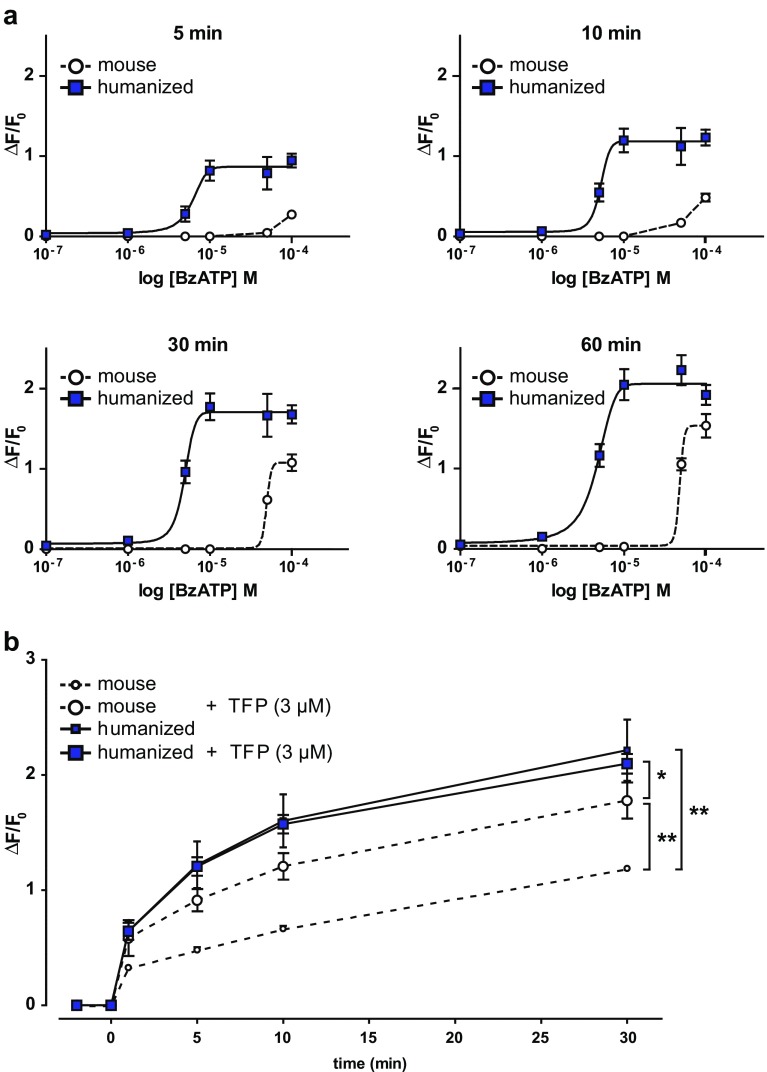
Comparison of BzATP-induced Yo-Pro-1 uptake of humanized and murine P2X7R expressing peritoneal macrophages. **a** Pore formation capacity of peritoneal macrophages expressing the murine and the humanized P2X7R. BzATP concentration-response curves for the mouse and humanized P2X7R following different incubation times. The humanized receptor shows higher sensitivity toward the agonist compared to the murine counterpart as determined by increasing concentrations of BzATP. **b** Peritoneal macrophages expressing the murine or humanized P2X7R show differential modulation of Yo-Pro-1 uptake by TFP upon concurrent stimulation with BzATP. Two-way RM-ANOVA + Bonferroni post hoc test, *p < 0.05, **p < 0.005, *n* = 3 wells per group, with cells obtained of 2 animals per genotype. Data are expressed mean ± SEM

We compared the functional properties of the humanized and mouse P2X7R using the Yo-Pro-1 uptake assay, which is based on the hallmark feature of P2X7R to form non-selective pores upon repeated or prolonged activation. The pore formation capacity measured by Yo-Pro-1 uptake was assessed in peritoneal macrophages derived from wild-type and humanized mice. Both receptor variants showed a positive correlation of Yo-Pro-1 uptake and BzATP concentration; however, the orthologs differed in their dynamics of Yo-Pro-1 uptake. BzATP concentration-response curves demonstrated a ~10-fold higher sensitivity of the humanized receptor which is activated at significantly lower levels of the agonist BzATP compared to the murine counterpart. While the murine receptor did not elicit any pore formation below 50 μM of BzATP, the humanized receptor induced pore formation at only 5 μM of agonist (Fig. 2a). Additionally, we observed species-specific differences of P2X7R with regards to its functional modulation by TFP. TFP is a common antipsychotic drug of the phenothiazine class. Based on our dose response experiments (Fig. 2a), we chose a BzATP concentration of 50 μM for this experiment. We confirmed the earlier observed differences between murine and humanized P2X7R with respect to Yo-Pro-1 uptake. This difference reached statistical significance 5 min after agonist application. Moreover, species-specific differences in response to TFP were detected between humanized and mouse P2X7Rs (Fig. 2b). A potentiating effect of TFP on Yo-Pro-1 uptake was exclusively detectable for the murine but not for the humanized P2X7R. This potentiating effect was observed throughout the time course, starting as early as 1 min after BzATP application (2-way RM-ANOVA: time F_4,5_ = 342.9, *p* < 0.0001; time × genotype F_4,5_ = 22.6, *p* < 0.005; genotype F_1,8_ = 28.2, *p* < 0.005; genotype × treatment F_1,8_ = 6.7, *p* < 0.05; Bonferroni post hoc test, *p* < 0.05). Taken together, our findings confirm previous results showing significant differences between human and mouse P2X7Rs with respect to agonist-dependent activation and modulation by pharmacological compounds.

### Establishment of P2X7R knockout mice

**Fig. 3:**
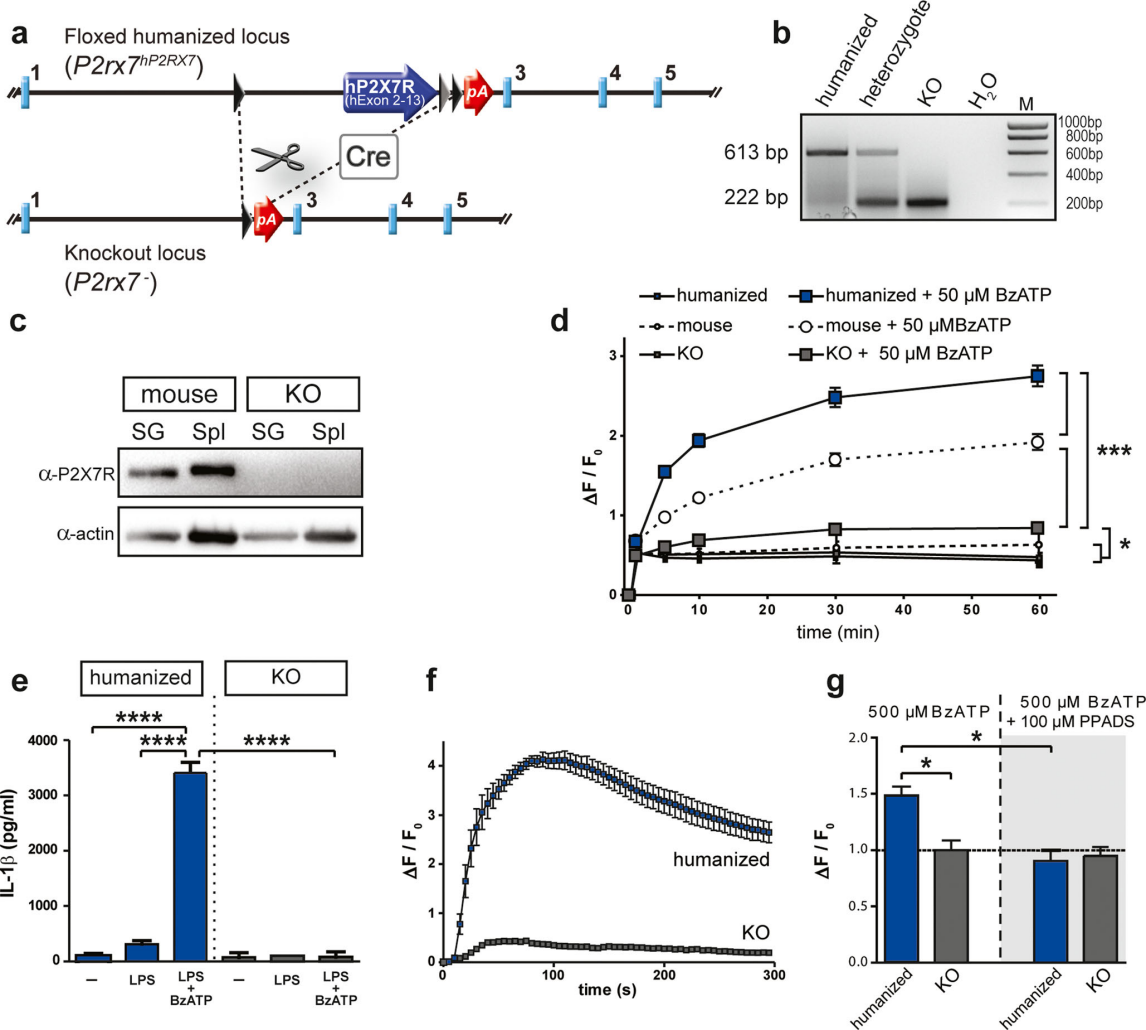
Generation and validation of P2X7R knockout mice. **a** The floxed humanized allele allows for Cre recombinase-mediated excision of the human P2RX7 resulting in a knockout (KO) allele. **b** Genotyping by PCR discriminates between humanized (613 bp) and KO allele (222 bp). **c** Representative Western blot demonstrating the loss of P2X7R protein in peripheral tissues of KO mice (SG, salivary gland; Spl, spleen). **d** Assessment of Yo-Pro-1 uptake shows that peritoneal macrophages obtained from KO mice lost their pore formation capacity compared to mouse or humanized cells upon prolonged stimulation of the receptor with 50 μM BzATP (two-way RM-ANOVA + Bonferroni post hoc test, *p < 0.05, ***p < 0.0005). **e** Functional validation of P2X7R KO as determined by interleukin-1β (IL-1β) release. Peritoneal macrophages of KO mice are no longer able to secrete IL-1β in response to LPS (3 μg/ml) stimulation and subsequent treatment with the 1 mM BzATP (two-way ANOVA + Bonferroni post hoc test, ****p < 0.0001). **f, g** Functional validation of P2X7R KO as indicated by lack of calcium uptake following stimulation of primary brain-derived cells of KO mice with 500 μM BzATP. **f** On the level of individual cells; **g** on the level of an entire cell population as determined by plate reader-based measurement (two-way ANOVA + Bonferroni post hoc test, *p < 0.05). Moreover, KO cells do not exceed calcium influx levels of cells treated with the purine receptor antagonist PPADS (100 μM). Data are expressed as mean ± SEM

**Fig. 4:**
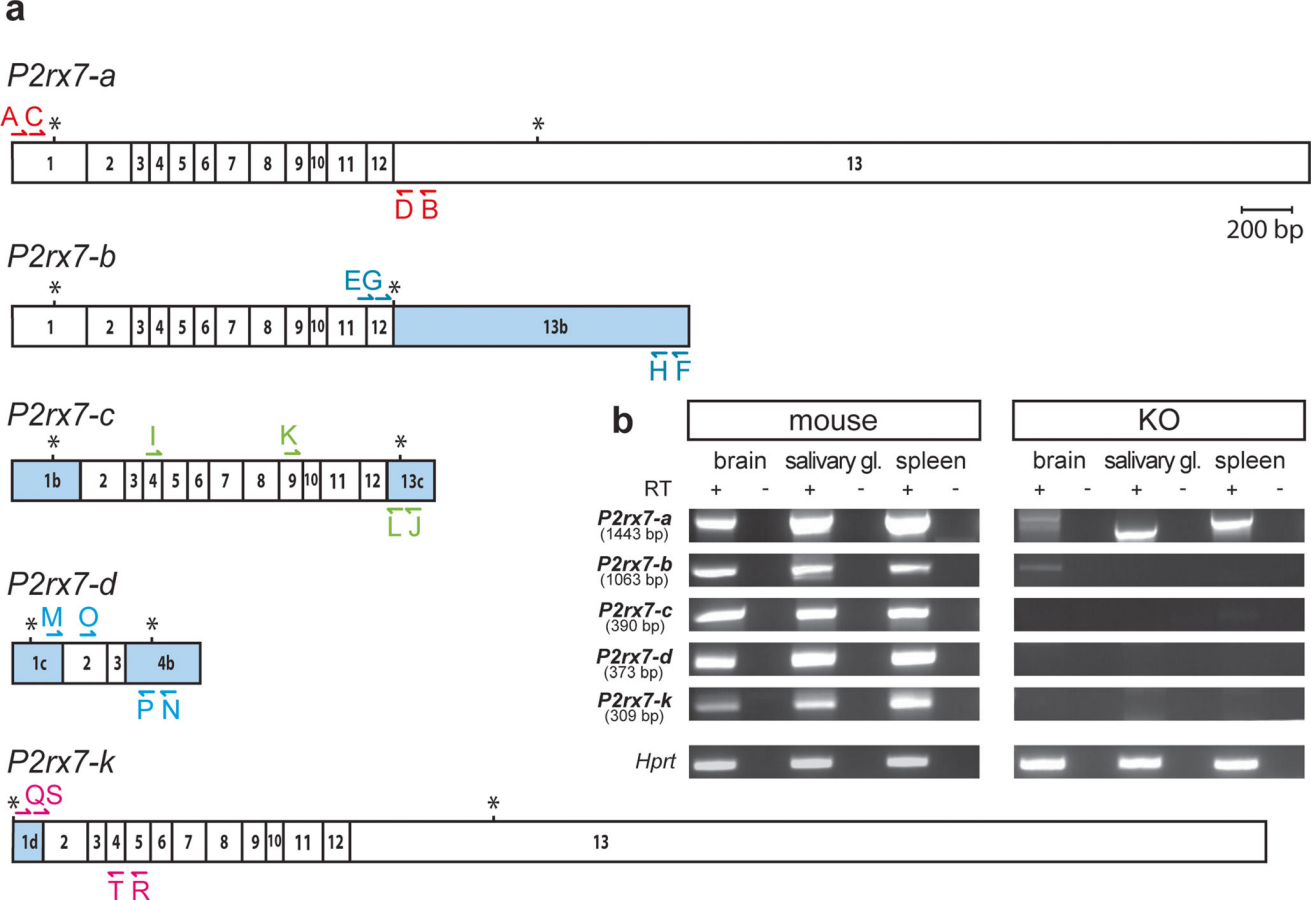
Assessment of *P2rx7* splice variants in P2X7R knockout mice. **a** Depicted are the 5 described *P2rx7* splice variants. Exons differing from the most abundant variant *P2rx7-a* are highlighted in *blue*. Translation start and stop sites are indicated by *asterisks*. Exon sizes are true to scale. Primers used for nested RT-PCR are schematically depicted above and below each transcript and are indicated by *capital letters and arrows*. **b** P2X7R knockout (KO) mice lack all *P2rx7* splice variants as determined by PCR. The remaining amplicons lack exon 2 and therefore do not represent transcripts translated to functional proteins

The humanized *P2rx7* locus was designed to enable Cre recombinase-mediated inactivation of hP2X7R expression without restoring murine P2X7R expression. To validate this novel conditional allele, we bred humanized *hP2RX7* mice to *Deleter-Cre* mice to generate a constitutive P2X7R KO mouse line (Fig. 3a, b). P2X7R protein expression was absent from the peripheral tissues of KO mice (Fig. 3c). The loss of receptor activity in P2X7R KO mice was further confirmed in the pore formation assay. Peritoneal macrophages obtained from KO animals were no longer able to show the characteristic Yo-Pro-1 uptake upon prolonged P2X7R stimulation. The response of P2X7R-deficient cells was only slightly increased compared to untreated cells (2-way RM-ANOVA: time F_5,14_ = 541.9, *p* < 0.0001; time × genotype F_10,30_ = 43.4, *p* < 0.0001; time × treatment F_5,14_ = 523.6, *p* < 0.0001; time × genotype × treatment F_10,30_ = 21.5, *p* < 0.0001; genotype F_2,18_ = 67.2, *p* < 0.0001; treatment F_1,18_ = 363.1, *p* < 0.0001; genotype × treatment F_2,18_ = 75.2, *p* < 0.0001; Bonferroni post hoc test, *p* < 0.05). As observed earlier, macrophages expressing humanized P2X7R showed an increased Yo-Pro-1 uptake compared to those expressing the murine receptor (Fig. 3d). Additionally, we observed that peritoneal macrophages of KO mice were no longer able to trigger the release of interleukin-1β (IL-1β) in response to LPS priming and BzATP stimulation (Fig. 3e, two-way ANOVA: genotype F_1,18_ = 169.82, *p* < 0.0001; treatment F_2,18_ = 137.92, *p* < 0.0001; genotype × treatment F_2,18_ = 139.27, *p* < 0.0001; Bonferroni post hoc test, *p* < 0.0001). Moreover, primary cells obtained from KO mouse brains showed a blunted response with regard to calcium uptake measured at the single cell level (Fig. 3f). The calcium uptake was comparable to cells treated with the purinergic receptor antagonist pyridoxalphosphate-6-azophenyl-2-4-disulfonic acid (PPADS). This suggests that a large proportion of the BzATP-induced calcium response is mediated by the P2X7R and can be blocked by the non-specific P2X receptor antagonist PPADS (Fig. 3g, two-way ANOVA: genotype F_1,12_ = 6.87, *p* < 0.02; treatment F_1,12_ = 14.05, *p* < 0.003; genotype × treatment F_1,12_ = 9.77, *p* < 0.009; Bonferroni post hoc test, *p* < 0.005). Finally, we assessed the presence of *P2rx7* splice variants in the KO mice. In contrast to previously published KO mouse lines, this null allele showed absence or functional disruption of all 5 known *P2rx7* splice variants in the brain and peripheral organs (Fig. 4). The residual transcripts detected in KO mice using primers for *P2rx7*-a (brain, salivary gland, and spleen) and *P2rx7*-b (brain) were specified by sequencing as non-functional splice products lacking exon 2, which is in accordance with the targeting strategy (Fig. 3a).

### P2X7R expression in different cell types of the central nervous system

**Fig. 5:**
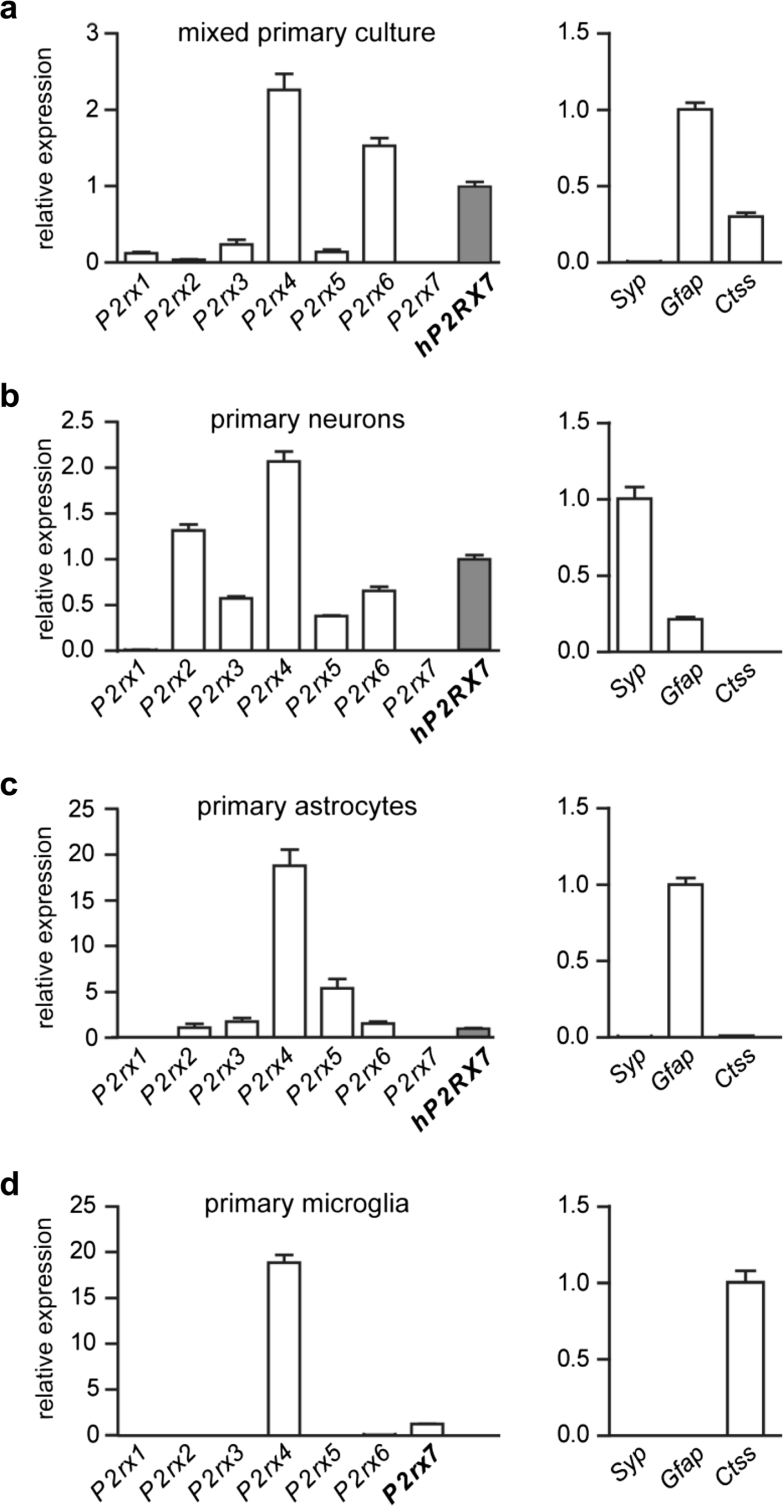
Expression of P2X receptor family members in different brain-derived primary cells. Relative mRNA expression levels of P2X receptor family members assessed in **a** mixed, **b** neuronal, **c** astrocytic, and **d** microglia primary cultures. The composition and purity of each culture were determined by the expression levels of cell type-specific markers (*right panels*). Humanized *P2RX7* mice were used for preparation of cultures except in **d** were wild-type mice were used instead. For each culture, *hP2RX7/P2rx7* acts as reference and is set to 1. For the graphs of the markers of the respective cell types (*right panel*), the highest expression is set as 1. Data are expressed as mean ± SEM

Next, we were interested in resolving the expression of P2X7R in the murine brain. First, we prepared primary cultures of different cell types derived from embryonic or early postnatal brains (hippocampus and cortex) of humanized or wild-type mice. Besides mixed cultures, we focused on specific neuronal, astrocytic, and microglial cultures from which we isolated RNA (Fig. 5). The purity of the cultures was monitored by analyzing the expression of cell type-specific markers for neurons (synaptophysin, *Syp*), astrocytes (glial fibrillary acidic protein, *Gfap*), and microglia (cathepsin S, *Ctss*) by RT-qPCR. Mixed cultures were mainly composed of astrocytes and microglia as indicated by the expression of *Gfap* and *Ctss*. Analyzing the expression of P2XR family members, we detected *hP2RX7*/*P2rx7* expression in all cultivated cell types. In all cultures, *P2rx4* showed the highest expression. With the exception of *P2rx1*, which showed the lowest mRNA levels in all investigated cultures, the expression level and pattern of the other family members varied between the different isolated and cultured cell types (Fig. 5). Taking into account that the culture conditions might directly affect P2X7R expression in primary cells, we additionally addressed its expression in vivo.

### P2X7R expression in the mouse brain

**Fig. 6:**
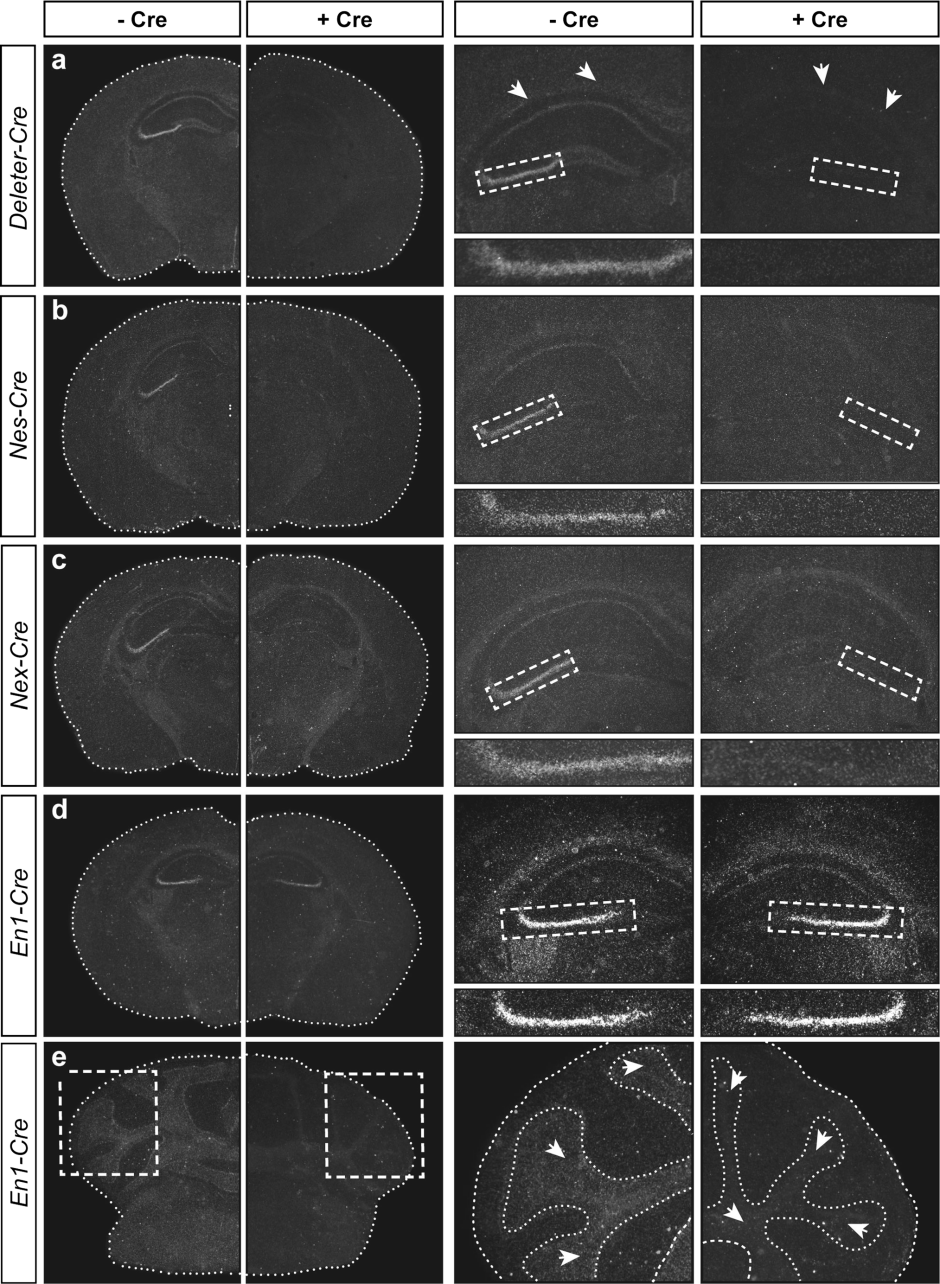
Analysis of P2X7R expression in the mouse brain by in situ hybridization using human- and mouse-specific riboprobes on conditional knockout mice. Depicted are coronal brain sections (overview, *left columns*) and magnifications (hippocampus or cerebellum, *right columns*) of control mice (−Cre) and respective conditional knockout mice generated by breeding to **a** *Deleter-Cre*, **b** *Nes-Cre*, **c** *Nex-Cre*, and **d, e** *En1-cre* mice (+Cre). *White arrows* indicate in situ hybridization signals in the white matter of the corpus callosum (**a**) and cerebellum (**e**)

To investigate expression levels in vivo, we took advantage of the conditional potential of our humanized *P2rx7* allele and bred *hP2RX7* mice to different tissue- and cell type-specific Cre drivers. In a first approach, we conducted in situ hybridization analyses to study P2X7R expression in more detail (Fig. 6). On brain sections from KO mice generated by breeding humanized mice, to *Deleter-Cre* mice, no mRNA of the human P2X7R was detectable demonstrating the specificity of the method (Fig. 6a). Of note is the loss of the faint ubiquitous signal, which was observed throughout the brain supporting a rather ubiquitous but weak expression of *P2rx7* mRNA. The neuron- and macroglia-specific *Nes*-*Cre* driver deleted the *hP2RX7* signal in the entire brain including the prominent signal in the CA3 of the hippocampus (Fig. 6b). By using the excitatory neuron-specific *Nex-Cre* driver, we confined the signal in the CA3 to neurons and even more specifically to excitatory, i.e., glutamatergic neurons (Fig. 6c). The *En1-Cre* driver, in which Cre recombinase is exclusively expressed in the mid-hindbrain area, enabled us to address the expression of P2X7R in neurons and macroglia in this brain region. As expected, *hP2RX7* expression was not affected in forebrain regions like the hippocampus (Fig. 6d). However, we observed strong differences in the hindbrain where the signal for *hP2RX7* was reduced in all regions of the cerebellum (Fig. 6f).

**Fig. 7:**
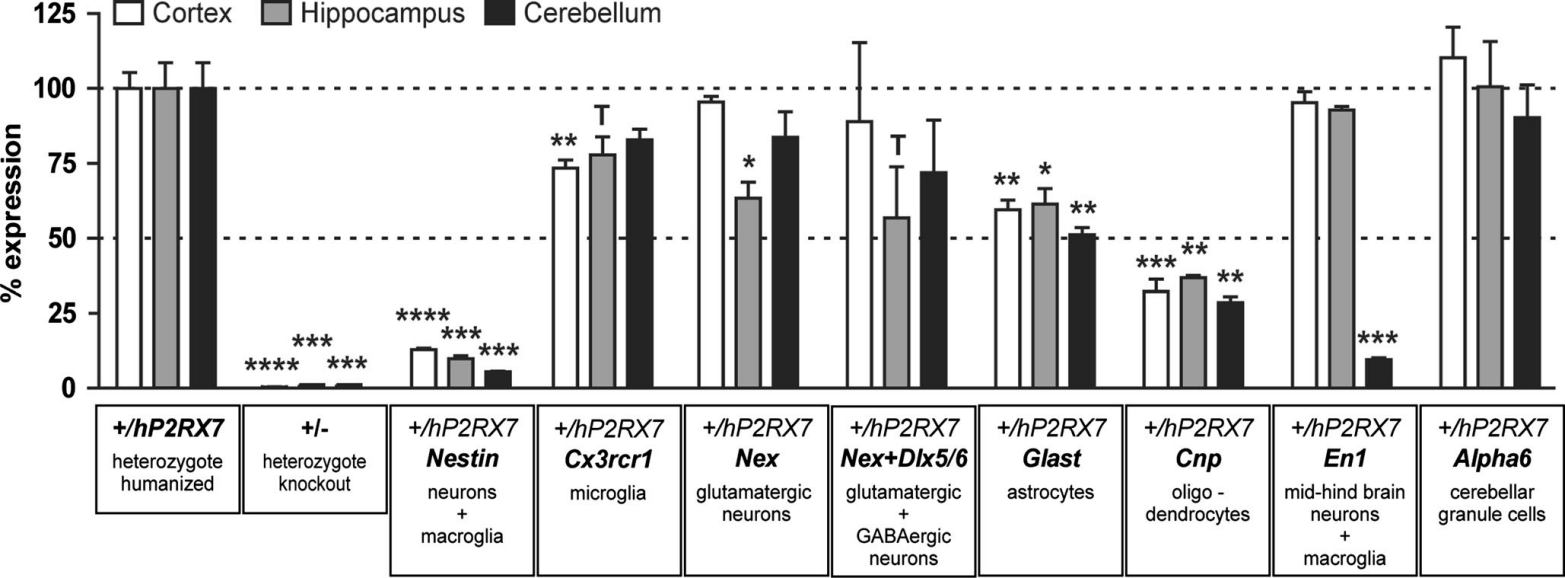
Analysis of P2X7R expression in the mouse brain by RT-qPCR using conditional knockout mice. Relative expression of *hP2RX7* was determined by real-time RT-qPCR using mRNA prepared from cortex, hippocampus, and cerebellum of heterozygous humanized mice (*P2rx7* ^*+/hP2RX7*^; +/hP2RX7). Expression of the floxed human *P2RX7* transcript was normalized to the expression of the endogenous murine *P2rx7* transcript (≙100 %). Heterozygous knockout mice demonstrated the specificity of the RT-qPCR. Cortex, hippocampus, and cerebellum of heterozygous mice positive for brain region- or cell type-specifically expressed Cre recombinase were analyzed accordingly. *t* test, *p < 0.05, **p < 0.005, ***p < 0.0005, ****p < 0.0001; significant effect compared to +/hP2RX7 for the respective brain region. T *p* < 0.1. Data are expressed as mean ± SEM (*n* = 3)

In situ hybridization is not informative in structures and cell types with low levels of P2X7R expression. Therefore, to gain a deeper understanding of where and to what extent P2X7R is expressed in the mouse brain, we conducted RT-qPCR analyses. To this end, we used heterozygous humanized mice carrying 1 wild-type and 1 conditional humanized *P2rx7* allele that were bred to different cell type-specific Cre drivers. We chose heterozygous mice in order to enable a normalization of the expression of the humanized *P2RX7* to the mouse *P2rx7* allele, which is insensitive to Cre recombinase-mediated inactivation. The expression of *hP2RX7* for each of the three investigated tissues (cortex, hippocampus, and cerebellum) in heterozygous humanized mice without Cre recombinase (*P2rx7* ^*+/hP2RX7*^) was set at 100 %. As shown before, the *hP2RX7* expression in heterozygous KO mice (*P2rx7* ^*+/−*^) was completely abolished or below the detection level (Fig. 7).

In *Nes-Cre* positive mice, the *hP2RX7* expression levels were decreased to 10–20 % in all investigated brain regions compared to *P2rx7* ^*+/hP2RX7*^ mice (*t* test: Ctx, t4 = 17.62, *p* < 0.0001; Hip, t4 = 10.65, *p* = 0.0004; Cb, t4 = 11.14, *p* = 0.0004). Since recombination in *Nes-Cre* mice, the recombination occurs in both neuronal and macroglial lineages but not in microglia; we additionally used *Cx3cr1-CreERT2* mice, which express Cre recombinase in the brain exclusively in microglia [47]. The *Cx3cr1-CreERT2* positive mice showed a 15–20 % reduction of *hP2RX7* expression in the brain (*t* test: Ctx, t4 = 4.76, *p* = 0.009; Hip, t4 = 2.14, *p* = 0.09; Cb, t4 = 1.9, *p* = 0.13). In line with the fact that *Nes-Cre* and *Cx3cr1-CreERT2* together cover almost all cell types found in the brain (with some exception, e.g., blood cells and cells forming the blood vessels), the combination of *hP2RX7* expression detected in both lines reached approximately 100 %.

To study the neuronal *P2RX7* expression in more detail, we used the *Nex-Cre* driver, which is specific for glutamatergic neurons. *Nex-Cre* positive mice showed the strongest reduction in *P2RX7* expression in the hippocampus (*t* test: Ctx, t4 = 0.9, *p* = 0.38; hip, t4 = 3.86, *p* = 0.012; Cb, t4 = 1.3, *p* = 0.25), which is in line with the in situ hybridization result. The remaining expression originates from other cell types which are also responsible for the overall weak in situ hybridization signal still present in *Nex-Cre* positive mice (Fig. 6c). A minor reduction in the cerebellum might originate from the partial recombination in deep nuclei and in the granule cell layer reported for this line [37]. In the cortex, no alteration in *hP2RX7* was observed. To cover almost all neurons, we included *Dlx5/6-Cre* mice, which exclusively recombine in GABAergic neurons (data not shown). However, combined recombination in excitatory and inhibitory neurons did not reduce *hP2RX7* expression any further (*t* test: Ctx, t4 = 0.4, *p* = 0.7; hip, t4 = 2.3, *p* = 0.09; Cb, t4 = 1.44, *p* = 0.22; Fig. 7).

Utilizing the *Glast*-CreERT2 driver, we could detect a substantial amount of *hP2RX7* expression in astrocytes in all analyzed brain regions (*t* test: Ctx, t4 = 6.86, *p* = 0.002; hip, t4 = 3.9, *p* = 0.018; Cb, t4 = 5.53, *p* = 0.005). Expression in oligodendrocytes was even higher, as revealed by the use of the oligodendrocyte-specific *Cnp*-Cre driver (*t* test: Ctx, t4 = 10.57, *p* = 0.0005; hip, t4 = 7.5, *p* = 0.0017; Cb, t4 = 8.2, *p* = 0.0012). Moreover, we could confirm the results from the in situ hybridization with respect to the expression of *hP2X7R* in the cerebellum by using the *En1*-Cre mice (*t* test: Ctx, t4 = 0.76, *p* = 0.49; hip, t4 = 0.84, *p* = 0.45; Cb, t4 = 10.64, *p* = 0.0004). Finally, the utilization of the *Alpha6-Cre* driver line excluded the possibility that P2X7R is expressed in granule cells—one of the main cell types in the cerebellum (*t* test: Ctx, t4 = 0.91, *p* = 0.41; hip, t4 = 0.033, *p* = 0.97; Cb, t4 = 0.70, *p* = 0.52).

## Discussion

The P2X7 receptor is a ligand-gated cation channel, which plays an important role in different physiological and pathophysiological processes. Alterations in receptor function caused by non-synonymous single nucleotide polymorphisms (SNPs) in the human *P2RX7* gene have been associated with various diseases including bone disorders, infectious disease, inflammatory and cardiovascular disorder, malignancies, and psychiatric disorders [48, 49]. The majority of non-synonymous SNPs are either loss- or gain-of-function mutations [50]. However, also primarily neutral polymorphisms might cause alterations in receptor activity, as we recently showed that co-expression of the neutral Gln460Arg polymorphism impairs P2X7R function when co-expressed with the wild-type variant [33]. Due to its involvement in health and disease, the P2X7R became an emergent target for the development of selective antagonists or modulators. Some of the oldest and still used antagonists are Brilliant Blue G (BBG) and PPADS [51, 52]. However, they were shown to lack full selectivity: BBG inhibits other receptors, e.g., pannexin 1 [53] whereas PPADS is able to affect other P2X receptor family members [54]. In recent years, novel and more specific P2X7R antagonists have been developed, and some have entered clinical trials [32, 55, 56]. Nevertheless, the in vivo characterization and evaluation of their therapeutic potential are mostly still pending. The well-known species-specific differences with regards to receptor sensitivity to agonists, antagonists, and modulators are complicating in vivo testing. Therefore, we generated a mouse model that expresses a humanized P2X7R under the control of the endogenous murine regulatory elements enabling the interrogation of the properties of the human P2X7R in vivo. We deliberately chose a strategy, which leaves the 5′ end of the murine *P2rx7* gene, including exon and intron 1, unaffected to ensure that the humanized receptor is expressed identically to the mouse P2X7R. This is fundamental prerequisite for the purposed determination of P2X7R expression in the mouse brain. Due to this strategy, we generated a chimeric P2X7R in which the vast majority of the receptor (553 aa) is of human origin while the first 42 aa are derived from mouse exon 1. Exon 1 encodes the intracellular N-terminus (30 aa), and the initial 12 aa of the first transmembrane domain. From the 11 aa that differ between human and mouse, there are 9 conservative substitutions. To the best of our knowledge, the two non-conservative substitutions in the intracellular domain (Trp-7-Cys and Thr-24-Met) as well as the conserved substitutions have not been demonstrated to affect receptor properties. Therefore, this mouse line is an animal model ideally suited to evaluate the properties of novel compounds targeting the human P2X7R including their therapeutic potential in vivo.

We used the Yo-Pro-1 uptake assay as a well-established readout for the assessment of P2X7R sensitivity toward different agonists and antagonists as well as for the comparison of receptor orthologs from different species [8, 9, 11]. All previous studies investigating inter-species differences of P2X7R orthologs were conducted in heterologously expressing cell lines. Here, we compared the Yo-Pro-1 uptake capacity of murine and humanized P2X7R endogenously expressed in primary cells obtained from respective mice. The detected difference in Yo-Pro-1 uptake between humanized and mouse P2X7R was comparable to previous reports [8]. To activate the pore formation via the murine P2X7R to levels comparable with the human P2X7R ~10 times higher BzATP concentrations were required. These observations indicate that the chimeric humanized P2X7R behaves largely similar compared to the pure human P2X7R. Nevertheless, further comprehensive testing is required to fully exclude differences in their properties. In addition, we observed that the modulator TFP had a potentiating effect on Yo-Pro-1 uptake exclusively on the murine receptor but not on the humanized P2X7R. Species-specific effects of positive and negative modulators have repeatedly been described for P2X7R orthologs [8, 10, 57, 58]. These findings suggest that our humanized mouse model is well-suited to discriminate properties of mouse and human P2X7R orthologs in an in vivo context and thereby opens new possibilities for the screening and evaluation of new P2X7R agonists and antagonists.

In addition, the humanized allele was designed to allow for Cre recombinase-mediated inactivation of the P2X7R. To date, 3 different P2X7R KO mouse lines and 1 knockdown line have been described [23, 24, 29, 30]. However, these lines have been shown to be flawed. In particular, the KO strategies applied in the lines from Pfizer [23] and GlaxoSmithKline [24] permit some splice variants evade inactivation [7, 12, 26–28]. Our novel KO line showed complete loss of receptor function by different readouts including Western blot, Yo-Pro-1 uptake, release of IL-1β, and uptake of Ca^2+^. Moreover, we specifically investigated the 5 known *P2rx7* splice variants and were able to demonstrate either their complete absence or their functional disruption. Thus, we believe that this conditional humanized allele shows a greater potential compared to previously generated KO alleles. To date, it is unclear to what extent the known phenotypes observed in existing KO mice are affected by the presence of residual *P2rx7* transcripts. Our novel KO allele provides the opportunity to critically reevaluate described phenotypes in a fully P2X7R negative background.

We used the mouse line expressing the human *P2RX7* transcript (exons 2–13) from the murine *P2rx7* locus as a sensitive reporter to address the controversially debated expression of P2X7R in the CNS [20, 31]. In a first step, we compared the mRNA expression of P2X receptor family members in primary cultures. We found that in all cases, *P2rx4* surmounts the other family members with regards to expression levels. The smallest difference between *P2rx4* and *P2rx7* expression was detected in neurons with around a 2-fold higher expression of *P2rx4*. In astrocytes and microglia, the difference is about 18-fold. This is an important finding considering that among all P2X family members, P2X4R is the closest relative of P2X7R [15]. Along these lines, the genes for both family members are located in close vicinity just 20–25 kb apart on human chromosome 12 and mouse chromosome 5, respectively. Moreover, P2X4R is up to 10 times more sensitive to the ligands ATP and BzATP than P2X7R [59]. It was further proposed that co-expressed P2X4 and P2X7 can form functional heteromers, although this finding has not been confirmed in more recent studies [60, 61]. Most importantly, however, is the finding that in vitro P2X7R is expressed in 3 of the main cell types of the brain: microglia, astrocytes, and neurons. Nevertheless, the expression of other family members, in particular P2X4R, has to be considered critically for functional analyses of the P2X7R in vivo.

Furthermore, we took advantage of the vulnerability of the humanized *P2rx7* allele to Cre recombinase-mediated inactivation. By breeding humanized mice to Cre driver mice, P2X7R expression was ablated in a region- or cell type-specific manner. Analysis of *hP2RX7* expression by in situ hybridization using a human-specific riboprobe revealed within the brain the hippocampal CA3 region as the most prominent expression domain. Expression in the CA3 subfield was further specified and specifically localized to soma of glutamatergic pyramidal neurons. Interestingly, the expression outside of the CA3 area is rather uniform but weak. Only the utilization of *Deleter-* and *Nes-Cre* mouse lines allowed us to ascertain that this is indeed a P2X7R-specific signal. Interestingly, the faint signal in *Nes-Cre* mice is slightly stronger than in *Deleter-Cre* mice providing some evidence for P2X7R expression in microglia, which have a different developmental origin than neurons or macroglia, and thus are not covered by the *Nes-Cre* driver. The RT-qPCR analysis clearly demonstrates expression of *hP2RX7* in the cortex and cerebellum, i.e., structures of low expression as detected by in situ hybridization. Using specific Cre drivers for astrocytes (*Glast-CreERT2*), oligodendrocytes (*Cnp-Cre*), and microglia (*Cx3cr1-CreERT2*) suggests that these cell populations are the sources for the low ubiquitous expression. Some more specific staining can be allocated to the white matter, e.g., in the corpus callosum and cerebellum that is probably related to oligodendrocytes. The RT-qPCR readily confirmed the expression of P2X7R in these main non-neuronal lineages of the CNS. The expression in astrocytes, oligodendrocytes, and microglia is in accordance with previous reports (reviewed in: [31]). However, this study demonstrates for the first time mRNA expression in all major cell lineages of the brain in a paralleled approach using mouse genetic tools thus avoiding any alterations in expression due to isolation and cultivation of primary cells. Based on the sensitivity of the method, we have strong evidence that neuronal expression is exclusively restricted to the hippocampal CA3 region. In the cortex and cerebellum of *Nex-Cre*, *Dlx-Cre*, and *Alpha6-Cre* positive mice, no reduction in *hP2RX7* was observed, arguing against neuronal P2X7R expression in these structures. Nevertheless, it cannot be fully ruled out that conditions exist that might induce P2X7R expression in neurons outside the hippocampus as it has been demonstrated for astrocytes and microglia [62]. In addition, the sensitivity of the applied methods has to be taken into account which might overlook low levels of P2X7R expression. Finally, it remains to be tested to what extent these results are transferable to the human brain considering that the transcriptional regulation might be different. In conclusion, our analyses at the mRNA level demonstrate that the P2X7R under basal conditions is rather ubiquitously expressed throughout major non-neuronal cell types of the mouse brain including astrocytes, oligodendrocytes, and microglia. A comparative quantification of P2X7R expression in these cell types is rather difficult since the actual contribution of each cell type to the total P2X7R expression depends on the numeric proportion of the respective cell type and the level of P2X7R expression therein. We unequivocally verified that P2X7R expression is restricted to glutamatergic neurons within the hippocampal CA3 region albeit with mRNA levels higher than in any other cell type of the brain as indicated by in situ hybridization.

Other means to interrogate the expression of a gene of interest are reporter and Cre mice [63]. *P2rx7-EGFP* reporter mice (Tg(P2rx7-EGFP)FY174Gsat) have been generated by the Gene Expression Nervous System Atlas (GENSAT) project (http://www.gensat.org/). These mice have been used to co-localize P2X7R-expressing cells in the brain with the transcription factor Sp1 [64]. In addition, this reporter line has been used to monitor P2X7R expression following a challenge such as status epileptics, which promotes enhanced green fluorescent protein (EGFP) expression in granule cells of the dentate gyrus [65] or conditions of ischemic tolerance, which induce expression of EGFP in microglia and activated astrocytes of *P2rx7-EGFP* mice [66]. The assumption that EGFP is reflecting endogenous expression is primarily based on the observed EGFP expression in macrophages and in the spleen—2 major sites of P2X7R expression [64]. In this context, it is of note that the strong expression at the mRNA level in the CA3 is not reflected by EGFP expression in this reporter mouse line (compare: http://www.gensat.org/). Recent reports on the variability even of bacterial artificial chromosome (BAC)-based transgenic mouse lines [67, 68] underscore the need for a more careful evaluation of the *P2rx7-EGFP* reporter line. Similarly, the KO allele generated by GlaxoSmithKline includes a LacZ reporter gene [24]. However, LacZ-staining of tissue sections of these mice revealed only staining in the ependymal cell layer and of cells in the submandibular gland [25]. Similarly, LacZ-staining of brain sections from *P2rx7* KO mice (*P2rx7* ^*tm1a(EUCOMM)Wtsi*^) generated by the EUCOMM program, which harbor a LacZ reporter cassette, did not reveal any staining (data not shown) supporting the generally low expression observed by in situ hybridization. In contrast to these single copy reporters, which were inserted in the endogenous gene locus, transgenic reporters harboring several copies of the construct might possess higher expression levels explaining the observations in *P2rx7-EGFP* mice. Alternatively, a *P2rx7*-specific Cre driver would provide the highest sensitivity and ultimately unravel the complexity of the P2X7R expression space. However, none of these reporter mice provide any information with respect to the subcellular localization of the receptor. Thus, mice expressing a P2X7R fused to a fluorescent reporter or equipped with a tag would be important to address its currently largely speculative subcellular localization in greater detail.

Taken together, in the present study, we established for the first time an animal model that enables the functional interrogation of the human P2X7R in the context of a mammalian model organism. We used this humanized mouse line to assess P2X7R expression and ultimately determined its distribution throughout the mouse brain and its main cell lineages. Moreover, this humanized mouse line provides a conditional allele that is sensitive to Cre recombinase-mediated inactivation. This null allele is superior to previously described KO alleles as it lacks any receptor activity and all known splice variants. Thus, this novel multifunctional allele provides the means to test compounds targeting the P2X7R under in vivo conditions and to address its function by more precise approaches since it avoids compensatory mechanisms and other caveats accompanying constitutive KO mice. Finally, taking into account the species-specific differences with respect to receptor sensitivity toward ligands, this humanized P2X7R mouse line could serve as an appropriate “wild-type” control for the in vivo interrogation of the numerous disease-associated non-synonymous SNPs in the human P2X7R.
